# Supplementary material for: Multi‐dataset identification of innovative feature genes and molecular mechanisms in keratoconus
Source: J Cell Mol Med. 2024 Sep 19;28(18):e70079. doi: 10.1111/jcmm.70079 (PMC11412914; doi:10.1111/jcmm.70079)
Supplement: Supplementary file 2 — Material S2. [file JCMM-28-e70079-s002.zip › 7.GSEA analysis/2.ATOH7/KEGG_ENDOCYTOSIS.html]

Details for gene set KEGG\_ENDOCYTOSIS[GSEA]

|  || Dataset | ATOH7.group.cls#h\_versus\_l |
| Phenotype | group.cls#h\_versus\_l |
| Upregulated in class | h |
| GeneSet | KEGG\_ENDOCYTOSIS |
| Enrichment Score (ES) | 0.24725796 |
| Normalized Enrichment Score (NES) | 1.3389413 |
| Nominal p-value | 0.118773945 |
| FDR q-value | 0.34764078 |
| FWER p-Value | 0.942 |
Table: GSEA Results Summary

  

Fig 1: Enrichment plot: KEGG\_ENDOCYTOSIS      
 Profile of the Running ES Score & Positions of GeneSet Members on the Rank Ordered List

  

| SYMBOL | TITLE | RANK IN GENE LIST | RANK METRIC SCORE | RUNNING ES | CORE ENRICHMENT || 1 | PIP5K1B | na | 2 | 1.293 | 0.0749 | Yes |
| 2 | SH3KBP1 | na | 106 | 0.762 | 0.1161 | Yes |
| 3 | ASAP1 | na | 1030 | 0.443 | 0.1151 | Yes |
| 4 | CXCR1 | na | 1036 | 0.442 | 0.1406 | Yes |
| 5 | GRK7 | na | 1255 | 0.414 | 0.1582 | Yes |
| 6 | CXCR4 | na | 1658 | 0.372 | 0.1681 | Yes |
| 7 | ERBB4 | na | 2108 | 0.336 | 0.1746 | Yes |
| 8 | PARD6B | na | 2333 | 0.321 | 0.1867 | Yes |
| 9 | CLTCL1 | na | 2354 | 0.319 | 0.2046 | Yes |
| 10 | EHD3 | na | 2993 | 0.281 | 0.2024 | Yes |
| 11 | SH3GL2 | na | 3214 | 0.269 | 0.2117 | Yes |
| 12 | PIP5K1A | na | 3253 | 0.267 | 0.2261 | Yes |
| 13 | SRC | na | 4033 | 0.235 | 0.2171 | Yes |
| 14 | ADRB3 | na | 4154 | 0.231 | 0.2270 | Yes |
| 15 | GRK4 | na | 4315 | 0.225 | 0.2354 | Yes |
| 16 | KDR | na | 4743 | 0.210 | 0.2352 | Yes |
| 17 | GRK1 | na | 5032 | 0.201 | 0.2385 | Yes |
| 18 | IQSEC3 | na | 5754 | 0.178 | 0.2280 | Yes |
| 19 | HSPA6 | na | 5975 | 0.172 | 0.2316 | Yes |
| 20 | RNF41 | na | 6072 | 0.169 | 0.2386 | Yes |
| 21 | MET | na | 6191 | 0.165 | 0.2448 | Yes |
| 22 | RABEP1 | na | 7175 | 0.139 | 0.2243 | Yes |
| 23 | RAB11FIP3 | na | 7345 | 0.135 | 0.2272 | Yes |
| 24 | EHD2 | na | 7408 | 0.133 | 0.2331 | Yes |
| 25 | AGAP2 | na | 8177 | 0.115 | 0.2176 | Yes |
| 26 | IL2RG | na | 8260 | 0.113 | 0.2218 | Yes |
| 27 | GRK5 | na | 8288 | 0.113 | 0.2275 | Yes |
| 28 | IL2RA | na | 8418 | 0.110 | 0.2302 | Yes |
| 29 | RAB11FIP5 | na | 8746 | 0.103 | 0.2267 | Yes |
| 30 | FGFR2 | na | 8886 | 0.101 | 0.2285 | Yes |
| 31 | SMURF1 | na | 9118 | 0.097 | 0.2274 | Yes |
| 32 | GIT2 | na | 9188 | 0.096 | 0.2310 | Yes |
| 33 | VPS45 | na | 9195 | 0.095 | 0.2363 | Yes |
| 34 | SMAP2 | na | 9440 | 0.091 | 0.2346 | Yes |
| 35 | ASAP2 | na | 9534 | 0.090 | 0.2371 | Yes |
| 36 | AP2A1 | na | 9542 | 0.090 | 0.2421 | Yes |
| 37 | PARD6A | na | 9543 | 0.090 | 0.2473 | Yes |
| 38 | CSF1R | na | 10042 | 0.081 | 0.2375 | No |
| 39 | SMURF2 | na | 10123 | 0.079 | 0.2398 | No |
| 40 | RAB5B | na | 10367 | 0.076 | 0.2371 | No |
| 41 | RAB11FIP2 | na | 10758 | 0.070 | 0.2299 | No |
| 42 | PSD3 | na | 10855 | 0.068 | 0.2311 | No |
| 43 | CLTB | na | 10927 | 0.067 | 0.2329 | No |
| 44 | VPS37A | na | 11113 | 0.064 | 0.2313 | No |
| 45 | CHMP5 | na | 11656 | 0.057 | 0.2189 | No |
| 46 | VPS37D | na | 11729 | 0.056 | 0.2201 | No |
| 47 | CHMP1B | na | 11804 | 0.055 | 0.2211 | No |
| 48 | KIT | na | 11952 | 0.053 | 0.2199 | No |
| 49 | ARFGAP3 | na | 12255 | 0.049 | 0.2140 | No |
| 50 | DNM1 | na | 12339 | 0.048 | 0.2144 | No |
| 51 | EGFR | na | 12485 | 0.046 | 0.2129 | No |
| 52 | MVB12B | na | 12578 | 0.045 | 0.2129 | No |
| 53 | EHD4 | na | 12811 | 0.042 | 0.2086 | No |
| 54 | ARRB2 | na | 12937 | 0.041 | 0.2074 | No |
| 55 | EHD1 | na | 13460 | 0.036 | 0.1943 | No |
| 56 | IL2RB | na | 13496 | 0.035 | 0.1954 | No |
| 57 | CHMP4C | na | 13533 | 0.035 | 0.1964 | No |
| 58 | RAB5A | na | 13630 | 0.034 | 0.1956 | No |
| 59 | TRAF6 | na | 13633 | 0.034 | 0.1975 | No |
| 60 | HLA-G | na | 13698 | 0.033 | 0.1976 | No |
| 61 | VTA1 | na | 13703 | 0.033 | 0.1994 | No |
| 62 | EPN1 | na | 13733 | 0.033 | 0.2005 | No |
| 63 | RAB11B | na | 13786 | 0.033 | 0.2008 | No |
| 64 | EGF | na | 13899 | 0.031 | 0.1994 | No |
| 65 | MVB12A | na | 13982 | 0.031 | 0.1988 | No |
| 66 | HSPA1L | na | 14016 | 0.030 | 0.1996 | No |
| 67 | DAB2 | na | 14119 | 0.029 | 0.1984 | No |
| 68 | CHMP4A | na | 14200 | 0.029 | 0.1977 | No |
| 69 | DNM3 | na | 14259 | 0.028 | 0.1977 | No |
| 70 | CHMP2A | na | 14312 | 0.027 | 0.1977 | No |
| 71 | PIP5K1C | na | 14534 | 0.025 | 0.1928 | No |
| 72 | HRAS | na | 14559 | 0.025 | 0.1936 | No |
| 73 | PLD1 | na | 14651 | 0.024 | 0.1924 | No |
| 74 | AGAP1 | na | 14727 | 0.023 | 0.1915 | No |
| 75 | ITCH | na | 14789 | 0.023 | 0.1911 | No |
| 76 | AP2B1 | na | 14815 | 0.023 | 0.1917 | No |
| 77 | PSD | na | 14861 | 0.022 | 0.1917 | No |
| 78 | SH3GL1 | na | 14876 | 0.022 | 0.1926 | No |
| 79 | HLA-A | na | 15242 | 0.019 | 0.1831 | No |
| 80 | HLA-C | na | 15354 | 0.018 | 0.1809 | No |
| 81 | SH3GL3 | na | 15637 | 0.016 | 0.1737 | No |
| 82 | TSG101 | na | 15648 | 0.016 | 0.1743 | No |
| 83 | DNM2 | na | 15705 | 0.015 | 0.1736 | No |
| 84 | ARF6 | na | 15942 | 0.013 | 0.1675 | No |
| 85 | RAB4A | na | 16409 | 0.010 | 0.1546 | No |
| 86 | AP2S1 | na | 16461 | 0.009 | 0.1536 | No |
| 87 | GIT1 | na | 16476 | 0.009 | 0.1537 | No |
| 88 | RBSN | na | 16532 | 0.008 | 0.1526 | No |
| 89 | GRK6 | na | 17178 | 0.003 | 0.1341 | No |
| 90 | PLD2 | na | 17242 | 0.002 | 0.1324 | No |
| 91 | VPS4B | na | 17279 | 0.002 | 0.1315 | No |
| 92 | ACAP1 | na | 17460 | 0.000 | 0.1263 | No |
| 93 | SH3GLB1 | na | 17480 | 0.000 | 0.1258 | No |
| 94 | STAM | na | 17504 | 0.000 | 0.1251 | No |
| 95 | USP8 | na | 17625 | -0.001 | 0.1217 | No |
| 96 | IQSEC1 | na | 17650 | -0.001 | 0.1211 | No |
| 97 | ADRB1 | na | 17706 | -0.002 | 0.1196 | No |
| 98 | CDC42 | na | 17874 | -0.003 | 0.1149 | No |
| 99 | RAB11A | na | 17896 | -0.003 | 0.1145 | No |
| 100 | WWP1 | na | 17964 | -0.004 | 0.1127 | No |
| 101 | CHMP2B | na | 18044 | -0.004 | 0.1107 | No |
| 102 | HLA-E | na | 18198 | -0.006 | 0.1066 | No |
| 103 | RUFY1 | na | 18321 | -0.007 | 0.1035 | No |
| 104 | VPS25 | na | 18380 | -0.007 | 0.1022 | No |
| 105 | HGS | na | 18392 | -0.007 | 0.1023 | No |
| 106 | AP2M1 | na | 18393 | -0.007 | 0.1028 | No |
| 107 | ARAP3 | na | 18434 | -0.008 | 0.1021 | No |
| 108 | IQSEC2 | na | 18717 | -0.010 | 0.0945 | No |
| 109 | LDLRAP1 | na | 18802 | -0.011 | 0.0927 | No |
| 110 | VPS37C | na | 19006 | -0.013 | 0.0876 | No |
| 111 | PRKCI | na | 19141 | -0.014 | 0.0845 | No |
| 112 | CCR5 | na | 19311 | -0.016 | 0.0805 | No |
| 113 | HLA-F | na | 19312 | -0.016 | 0.0814 | No |
| 114 | ERBB3 | na | 19354 | -0.016 | 0.0812 | No |
| 115 | SNF8 | na | 19360 | -0.016 | 0.0820 | No |
| 116 | ARFGAP2 | na | 19372 | -0.016 | 0.0826 | No |
| 117 | EPN2 | na | 19391 | -0.016 | 0.0830 | No |
| 118 | VPS4A | na | 19528 | -0.017 | 0.0800 | No |
| 119 | VPS28 | na | 19723 | -0.019 | 0.0755 | No |
| 120 | CLTA | na | 19774 | -0.020 | 0.0752 | No |
| 121 | CHMP6 | na | 19791 | -0.020 | 0.0759 | No |
| 122 | CBLC | na | 20019 | -0.022 | 0.0706 | No |
| 123 | NEDD4 | na | 20241 | -0.024 | 0.0656 | No |
| 124 | PARD3 | na | 20314 | -0.025 | 0.0650 | No |
| 125 | CHMP3 | na | 20442 | -0.026 | 0.0628 | No |
| 126 | ACAP2 | na | 20528 | -0.027 | 0.0619 | No |
| 127 | TFRC | na | 20531 | -0.027 | 0.0634 | No |
| 128 | EEA1 | na | 20860 | -0.030 | 0.0557 | No |
| 129 | PARD6G | na | 20864 | -0.030 | 0.0573 | No |
| 130 | PRKCZ | na | 21192 | -0.034 | 0.0498 | No |
| 131 | HSPA1A | na | 21593 | -0.038 | 0.0405 | No |
| 132 | ACAP3 | na | 21698 | -0.039 | 0.0397 | No |
| 133 | RAB31 | na | 21753 | -0.040 | 0.0405 | No |
| 134 | PSD4 | na | 21961 | -0.042 | 0.0370 | No |
| 135 | PIKFYVE | na | 22283 | -0.046 | 0.0303 | No |
| 136 | HSPA1B | na | 22392 | -0.047 | 0.0300 | No |
| 137 | RAB22A | na | 22602 | -0.050 | 0.0268 | No |
| 138 | HLA-B | na | 23071 | -0.056 | 0.0165 | No |
| 139 | SH3GLB2 | na | 23329 | -0.059 | 0.0125 | No |
| 140 | ADRB2 | na | 23374 | -0.060 | 0.0147 | No |
| 141 | RAB11FIP1 | na | 23524 | -0.062 | 0.0140 | No |
| 142 | FGFR4 | na | 23580 | -0.063 | 0.0161 | No |
| 143 | RAB5C | na | 23680 | -0.065 | 0.0170 | No |
| 144 | VPS36 | na | 23682 | -0.065 | 0.0207 | No |
| 145 | PIP4K2B | na | 23746 | -0.066 | 0.0227 | No |
| 146 | STAMBP | na | 23993 | -0.070 | 0.0196 | No |
| 147 | DNM1L | na | 24243 | -0.073 | 0.0167 | No |
| 148 | MDM2 | na | 24258 | -0.073 | 0.0205 | No |
| 149 | CHMP4B | na | 24437 | -0.076 | 0.0198 | No |
| 150 | HSPA2 | na | 24524 | -0.077 | 0.0218 | No |
| 151 | STAM2 | na | 24567 | -0.078 | 0.0251 | No |
| 152 | CBL | na | 24630 | -0.079 | 0.0279 | No |
| 153 | FGFR3 | na | 24695 | -0.080 | 0.0307 | No |
| 154 | VPS37B | na | 24799 | -0.082 | 0.0325 | No |
| 155 | ARFGAP1 | na | 24933 | -0.084 | 0.0335 | No |
| 156 | NTRK1 | na | 25343 | -0.091 | 0.0269 | No |
| 157 | EPN3 | na | 25349 | -0.091 | 0.0321 | No |
| 158 | HSPA8 | na | 25938 | -0.103 | 0.0210 | No |
| 159 | SMAP1 | na | 25988 | -0.104 | 0.0256 | No |
| 160 | PSD2 | na | 26147 | -0.107 | 0.0272 | No |
| 161 | LDLR | na | 26673 | -0.118 | 0.0189 | No |
| 162 | NEDD4L | na | 26737 | -0.120 | 0.0240 | No |
| 163 | AP2A2 | na | 27123 | -0.128 | 0.0203 | No |
| 164 | IGF1R | na | 27632 | -0.142 | 0.0138 | No |
| 165 | FLT1 | na | 27682 | -0.143 | 0.0207 | No |
| 166 | PDCD6IP | na | 28270 | -0.158 | 0.0128 | No |
| 167 | ARAP1 | na | 28432 | -0.163 | 0.0176 | No |
| 168 | EPS15 | na | 28913 | -0.176 | 0.0139 | No |
| 169 | ASAP3 | na | 29010 | -0.180 | 0.0216 | No |
| 170 | ARRB1 | na | 29554 | -0.196 | 0.0172 | No |
| 171 | RET | na | 29684 | -0.200 | 0.0251 | No |
| 172 | DNAJC6 | na | 29708 | -0.200 | 0.0360 | No |
| 173 | F2R | na | 29759 | -0.202 | 0.0463 | No |
| 174 | RAB11FIP4 | na | 29962 | -0.209 | 0.0526 | No |
| 175 | CXCR2 | na | 30340 | -0.223 | 0.0546 | No |
| 176 | ARAP2 | na | 30960 | -0.246 | 0.0509 | No |
| 177 | CLTC | na | 31200 | -0.254 | 0.0588 | No |
| 178 | CBLB | na | 31517 | -0.270 | 0.0652 | No |
| 179 | PDGFRA | na | 33905 | -0.477 | 0.0238 | No |
Table: GSEA details [plain text format]

  

Fig 2: KEGG\_ENDOCYTOSIS      
 Blue-Pink O' Gram in the Space of the Analyzed GeneSet

  

Fig 3: KEGG\_ENDOCYTOSIS: Random ES distribution      
 Gene set null distribution of ES for **KEGG\_ENDOCYTOSIS**
